# Knowledge, Attitude, and Practice of Physiotherapists about Cardiac Rehabilitation Program Adherence among Patients Discharged from the Hospital after Cardiac Surgery in India

**DOI:** 10.1155/2024/8825476

**Published:** 2024-05-17

**Authors:** Ayman Gondekar, Vijay Pratap Singh, Stephen Rajan Samuel, Harish Raghavan, Bidita Khandelwal, K. Vijaya Kumar

**Affiliations:** ^1^Department of Physiotherapy, Kasturba Medical College, Mangalore, India; ^2^Manipal Academy of Higher Education, Manipal, India; ^3^Cardiothoracic and Vascular Surgeon, Kasturba Medical College Hospital, Mangalore, India; ^4^Department of Medicine, Sikkim Manipal University, Sikkim Manipal Institute of Medical Sciences, Gangtok, India

## Abstract

**Background:**

In most settings, patients receive phase 1 cardiac rehabilitation in CTVS ICU at the hospital, but there are several barriers to follow-up after patients are discharged from the hospital. Physiotherapists play an important role in the enrolment and continuation of cardiac rehabilitation. Thus, we aim to study the knowledge, attitude, and practice of physiotherapists about CR program adherence among patients discharged from the hospital after cardiac surgery.

**Objectives:**

(i) To study the knowledge of physiotherapists about the importance of cardiac rehabilitation after discharge; (ii) to know the attitude of physiotherapists towards cardiac surgery patients after discharge; and (iii) to know what approach various centres are applying for patients after discharge to ensure adherence to cardiac rehabilitation.

**Methods:**

A questionnaire was developed with reference to the objectives of the study, which was answered by a total of 127 physiotherapists.

**Results:**

The overall response rate was 42.3%; nearly 35.4% of the participants indicated that they knew a lot about CR, while 5.5% said they knew very little. Regarding the program's content, 36.2% of participants reported having a medium degree of awareness of the diverse CR components, while 8.6% reported having very little knowledge of them. Only about one-third, 35.7% stated that CR in India is effective and 95% believed that CR will have an added value for the country. Approximately 80% of respondents thought that it would be challenging for a physiotherapist to recommend patients to a CR in the nation. Nearly 35% of respondents believed that they, “themselves as physios,” needed to commence CR, and slightly less than 70% thought that doctors were required to choose and refer the patients when asked who should take the initiative to start this kind of programme in the country. A little over 40% of respondents said that insurance firms are also involved in starting a CR programme.

**Conclusion:**

Physiotherapists have good knowledge of cardiac rehabilitation. However, their attitude and practice towards adherence to exercise protocols are confounded by various clinician- and patient-level factors.

## 1. Introduction

Cardiovascular disease (CVD) is a leading cause of morbidity and mortality around the world, including India [1]. “According to the World Health Organization, a group of disorders of the heart and blood vessels include coronary heart disease, cerebrovascular disease, rheumatic heart disease, and other conditions” [2]. CVDs are a leading cause of hospitalization which leads to loss of work and function. There are various modifiable and nonmodifiable predisposing factors of CVDs on a biological, behavioural, and social level, some of which are lack of physical activity, smoking, consumption of alcohol, unhealthy diet, overweight or obesity, hypertension, diabetes, hyperlipidaemia, stress, and genetic predisposition.

Phases 2 and 3 of cardiac rehabilitation are an outpatient program for secondary prevention that include exercise training, which is structured, as well as counselling and education [1]. CR is delivered using a multidisciplinary approach that includes assessment of the patient, management of risk factors, nutritional counselling, psychosocial therapy, physical activity counselling, and exercise. Exercise is an important component of CR, and thus, physiotherapists play a vital role in prescribing tailored exercise programs for individuals. Various studies have already proved the benefits of exercise-based CR in reducing cardiac mortality, morbidity, and hospitalization [3]. International guidelines recommend that all patients with cardiovascular disease, coronary heart disease, heart failure, arrhythmias, congenital heart disease, and valvular heart disease should be referred to CR as a vital component of care. Additionally, CR should be made available to everyone, regardless of their age, sex, ethnicity, and clinical condition [4]. Despite strong evidence of benefit, support from national and international recommendations [5], and decades of work to increase patient engagement, CR continues to be underutilized internationally [6]. Even with the program's established benefits, it has several hurdles in its implementation, including a low level of participation, gender-biased participation and referrals, adherence and [2] drop-out issues, and management of resources. Low referrals, distance from the hospital, lack of enthusiasm, and the unwillingness of the patients to participate are some of the causes for low participation [7, 8].

In most of the settings, patients receive phase I cardiac rehabilitation (CR) postcardiac surgery under the direct supervision of physiotherapists in CTVS ICU. Previous studies have shown that there are several barriers to follow-up after patients are discharged from the hospital [1]. Home-based cardiac rehabilitation was introduced to widen its access and participation [9–11]. Most of the patients receive a home-based cardiac rehabilitation program as a part of their phase II CR which is unsupervised. There is limited evidence about the supervision of these patients in phase II CR and hence their adherence to the phase II CR program when they are at home. Whether the patient participates in a supervised centre-based or a home-based program largely depends on the local availability and preferences of the individual patient [9]. Few reports exist on the enrolment and adherence rates of patients for maintenance cardiac rehabilitation (MCR) due to the lack of standardization of this service [12]. However, few studies have revealed that the intervention deliverer and the delivery format play a role in increasing the enrolment of patients [13]. There are very limited centres in India who call their patients for phase II CR under the supervised program at the hospital. Moreover, only a limited number of physicians and cardiologists in India appreciate the benefits of CR. Even when the patients are called, their attendance is limited by barriers such as transportation, low socioeconomic status, nonreferral from surgeons and physicians, the need to return to work, and insurance barriers [12]. Physiotherapists play an important role in the enrolment and continuation of cardiac rehabilitation by patients.

Thus, we aim to study the knowledge, attitude, and practice of physiotherapists about CR program adherence among patients discharged from the hospital after cardiac surgery.

## 2. Methodology

Utilising a questionnaire that was organised and self-administered, a quantitative cross-sectional approach was chosen. Participants were Indian physiotherapists. The institutional research committee KMC, Mangalore, and the institutional ethics committee, Protocol no. IEC KMC MLR 01/2022/35, gave their consent for the conduct of this survey. The selected participants were sent the validated questionnaire via e-mail. Among them was an Indian physical therapist. The study's participants received no compensation for their participation. Those who did not respond even after the second reminder were eliminated from the study. Two reminders regarding the same were sent at intervals of one month. Participants were included using a method of systematic random sampling. A total of 127 of the 300 participants returned a completed survey. The self-administered questionnaire was created using a review of the literature. Experts in the field, including the healthcare team, fitness and nutrition specialists, physical therapists, and counsellors from KMC, Mangalore, verified this questionnaire. They provided feedback, and the questionnaire was revised accordingly. There were 27 questions in the survey, which was based on the three parts of the WHO's KAP framework.

The questionnaire was divided into 5 parts:

1. Part 1 covered questions on knowledge of physiotherapists about CR
2. Part 2 explored the attitudes of physiotherapists on CR
3. Part 3 assessed the daily practices of physiotherapists on CR
4. Part 4 investigated the barriers faced by physiotherapists when referred patients to CR
5. Part 5 studied the sociodemographic data of the responders.

The survey was confidential. Each participant gave their written agreement prior to the start of the questionnaire. Prior to the survey, the question wording was tested. The survey used the English language version of the questionnaire. For following data collection and screening, GraphPad Prism was used for data entry, cleaning, and analysis. Descriptive statistics, including frequencies, means, medians, standard deviations, and percentages, were used in the study for each response variable.

### 2.1. Inclusion Criteria

Participants should have a minimum qualification of Bachelor of Physiotherapy, participants should have a minimum of three years of experience in CR after completion of their education, participants should be affiliated to cardiac centres or should have an independent clinic of physiotherapy, and participants should have experience in inpatient, outpatient, and home-based CR programs.

### 2.2. Exclusion Criteria

The exclusion criterion was the participants other than the Indian nationality.

## 3. Results

### 3.1. Content Validation

A criterion of 60% and above was kept to include the question in the questionnaire of the study. All experts graded 4 or 5 for the 27 questions, which ranked greater than 60%. The validity of the questionnaire was determined using the content validity index (CVI). The item-content validity index (I-CVI) and scale-content validity index (S-CVI) were calculated for the item pool. The S-CVI was calculated by taking the average I-CVI and dividing it by the total number of items. The S-CVI was 0.97. From the values obtained, we could conclude that the questionnaire had achieved a satisfactory level of content validity.

In total, 127 questionnaires were gathered among the 300 participants in the conference. Thus, the overall response rate was 42.3%. Results are shown in the following tables.

### 3.2. Sociodemographic Characteristics

MPT was the greatest degree of training for 62 (40.8%) responders. Only 4 (3.1% of responders) had a Ph.D., whereas 61 (48%) were BPTs. The majority of respondents, 61 (48.0%), either worked in a hospital or an educational/research institute. 5 respondents (3.9%) reported having a community service job. The majority of participants were employed in cities. 31 (24.4%) and 86 (67.7%) of them had suburban jobs. Only 10 (7.9%) people held jobs in remote areas. 29 (22.8%) and 98 (77.2%) of the physiotherapists were men, respectively. The work experience of the respondents varied as shown in Table 1. The majority of the participants, 80 (63.0%), had experience of between three and six months, while seven (5.5%) had experience of between six and twelve months, ten (7.9%) had experience of between one and two years, and four (3.1%) had experience of more than five years.

### 3.3. Knowledge about CR

Table 2 presents the results on the level of knowledge about CR. The table reveals that nearly a third (35.4 percent, *n* = 45) of the participants indicated that they knew a lot about CR, while 5.5% (*n* = 7) said they knew very little. Regarding the program's content, 36.2% of participants (*n* = 46) reported having a medium degree of awareness of the diverse CR components, while 8.6% (*n* = 11) reported having very little knowledge of them.

### 3.4. Attitude towards CR

Our results also provide information on the attitude towards CR. Most of the respondents in our survey had a positive attitude towards CR. Only about one third, 35.7% (*n* = 115), stated that CR in India is effective and 95% (*n* = 306) believed that CR will have an added value for the country. In total, 95.7% (*n* = 308) of the respondents agreed that CR could improve the quality of life and lifestyle of a stable patient postsurgery or suffering from cardiovascular and pulmonary diseases. In addition, 95.3% (*n* = 307) indicated that CR could change patient behaviour postsurgery or suffering from cardiovascular diseases (CVDs). Further details can be found in Table 3.

### 3.5. CR Practice among Physiotherapists

Our findings reveal the respondents' opinions on the types of patients who should be qualified to begin a CR in relation to the practice of CR. According to the majority of respondents, patients with NCDs (postcardiac event, cardiac surgery, COPD patients, or those with pulmonary disorders) should be “eligible candidates” for CR. Further details on this issue can be found in Table 4. Approximately 80% of respondents thought that it would be challenging for a physiotherapist to recommend patients to a CR in the nation. Nearly 35% of respondents believed that they, “themselves as physios,” needed to commence CR and slightly less than 70% thought that doctors were required to choose and refer the patients when asked who should take the initiative to start this kind of programme in the country. A little over 40% of respondents said that insurance firms are also involved in starting a CR programme. The majority of respondents, nearly 50% thought that policy makers, those who created the policy, should play a significant part in integrating the patient into a CR programme. Further studies revealed that PT conducted hardly any CR-related tasks at their workplaces or patients' homes in the previous month. Particularly, more than 80% of the respondents stated that they had not seen any patients with cardiopulmonary illnesses over the preceding month at their place of employment, in a private practice, or in a hospital setting. Additionally, over eighty percent of respondents said that they did not treat patients with cardiopulmonary disorders in their homes. A total of 63.7% (*n* = 81) of the PT in our study, only “1 to 2 patients suffering from cardiopulmonary diseases” received medical care at hospitals or private clinics.

### 3.6. Barriers Faced by Physiotherapists

Our survey produced information on the types of impediments and whether the PT encounters them when integrating a CR programme in India. When patients are directed to rehabilitation treatment by doctors or other primary care providers, over 60 percent of respondents mention potential impediments. One out of two respondents believes that the primary obstacle is a lack of expertise in putting a CR programme into place. The scarcity of experts in this subject is the second most commonly cited hurdle. The unavailability of specialised centres in India is the third major obstacle. When patients are referred by doctors to begin a programme, slightly more than 20% of respondents cited the lack of patient enthusiasm in CR as a barrier. Additionally, more than 75% of respondents believe that a CR programme will not help patients and will not alter their behaviour (details can be found Table 5).

Additionally, more than half of the respondents acknowledged that they would encounter financial obstacles when patients were referred by primary healthcare providers to begin treatment, and they believed that the lack of insurance coverage for the programme was a barrier to entry into the CR programme. Over fifty percent of the respondents regarded a lack of physician support as the greatest barrier to starting a CR programme. The need for more training for physiotherapy students, increased awareness of the value of CR, and the implementation of a master's degree in this field of practice, which is lacking in the nation, are other barriers that one-fourth of the participants suggest fall under the category “others.”

### 3.7. Role Played by Physiotherapists in Prevention through CR

According to our responders, physical therapy is essential in the fight against NCDs. More than 90% of respondents agreed with this assertion. The majority of respondents concurred that physical therapists (PTs) have additional important prevention roles, such as educating patients about the advantages of leading healthy, active lifestyles and promoting daily physical activity in their clinical practice (beyond therapeutic exercises) to advance the prevention of cardiovascular and pulmonary diseases. The majority of PTs, according to research, are assured when advising patients on physical therapy and serve as role models for their patients. Regarding CR programmes, PTs are eager to take part in the evaluation of patients' body mass index (BMI), the 6-minute walk test to determine exercise ability, the screening of cardiovascular risk factors, and the provision of patient education, dietary advice, and smoking cessation counselling. Last but not least, physical therapists (PT) are crucial to the patient's psychological management during treatment (Table 6).

### 3.8. Support for Types of CR Delivery and Recommendations by Physiotherapists

Our data indicate that PT in India supports all types of CR programmes in the nation, including telemedicine delivery and inpatient and outpatient clinical settings (Table 7). The majority of respondents are in favour of setting up CR centres nationwide as inpatient or outpatient clinics as well as the potential use of telerehabilitation in the future. In our study, PT made a number of suggestions for CR in India. The participants specifically suggested that physicians receive additional training and instruction in CR to increase awareness of the field's significance in the nation. They also emphasised the need to find a solution to the money problem of paying for patients' enrolment in CR. Additionally, they suggested promoting CR for the prevention of NCDs and removing obstacles to patient enrolment in CR.

## 4. Discussion

Our study aimed to assess the knowledge, attitude, and practice of physiotherapists about cardiac rehabilitation programme adherence among patients discharged from the hospital after cardiac surgery. The questionnaire was sent to physiotherapists all over India but was answered by 127 physiotherapists out of 300, mostly from the western and southern zones of India. This indicates that 42% of the participants responded to the questionnaire. The highest qualification of the physiotherapists was either Ph.D., and most were working in an educational/research institute or a hospital. The average work experience of the physiotherapists who responded was ∼6 months, and all considered cardiac rehabilitation to be important or very important. However, according to many responders, it was not a common practice to recommend or prescribe cardiac rehabilitation in their place of practice. Moreover, the maximum number of physiotherapists had not received any formal training for cardiac rehabilitation other than their BPT-level physiotherapy curriculum. Many claimed to have a multidisciplinary team at their centre for cardiac rehabilitation, but not all actively participated as a team member.

Our questionnaire included questions assessing the knowledge of the therapists with respect to cardiac rehabilitation. Most of the respondents answered correctly that cardiac rehabilitation has 3-4 phases. On the question where is the phase 1 CR given, all participants correctly answered “at the hospital” and that patients should be followed up after discharge. Most physiotherapists considered phase 2 cardiac rehabilitation to be equally important as phase 1. But many believed that phase 3 cardiac rehabilitation does not require follow-up and did not consider cardiac rehabilitation to be a lifelong program, which is a wrong conception [14, 15]. This shows that physiotherapists had enough knowledge to appreciate the benefits of phase 2 CR and they valued phase 2 equally. There have been studies in the past which have assessed the knowledge and attitude of cardiologists and physicians towards CR [1, 16], but though physiotherapists are providers of CR, there is limited evidence of assessment of the knowledge and attitude of physiotherapists towards CR.

Our findings are in line with what we expected and are backed up by related research. In particular, we discover that Indian physiotherapists value their contribution to CR. The significance of the function played by the PT in the promotion of CR has been shown by numerous studies. The role of PT in bridging the gap between patients and other healthcare providers has been supported by a number of findings, is advocated by worldwide guidelines and WHO, and is underlined by our results [17]. PTs are significant contributors to the primary care team as vital providers of high-quality healthcare due to their credentials and capacity to screen, diagnose, and prescribe the proper treatment or referral [18].

As shown in our findings, physiotherapists think that CR has the potential to enhance patients' quality of life. Extensive exercise-based rehabilitation programmes have been proven effective on a global scale, and these programmes are now widely recognised as being crucial in the treatment of pulmonary and cardiovascular disorders. All causes of mortality have been demonstrated to be greatly reduced by CR [19, 20]. CR can improve mental well-being for patients with NCDs, improve medical outcomes, facilitate a quick return to work, and foster the growth of self-management abilities [21–25]. Overall, when combined with drug therapy, CR is one of the most affordable treatments for pulmonary and CVDs [26].

Our findings demonstrate that Indian physiotherapists promote physical activity outside therapeutic exercises in their regular practice. Our analysis highlights a favourable attitude towards the establishment of future CR centres in the nation. PT agrees that the country will benefit from CR centres. According to the results of our poll, physical therapy helps patients lead healthy lives after accidents or surgeries, as reported by the respondents.

Finally, given that PT is essential for the emergence and expansion of CR, our findings are consistent with previous research. Numerous research studies conducted in the UK, Australia, Cyprus, Greece, and the USA corroborated this. These studies reaffirm the significance of CR programmes. According to a study by Karam Turk-Adawi, there is a dearth of CR programmes worldwide; just 38.8% of nations have them. Particularly, 68.0% of high-income nations and 23% of LMICs (28.2% of middle-income countries and 83% of low-income countries) have CR. Estimates of CR density ranged from 1 programme per 01 to 64 million people [27–30].

As part of a multidisciplinary team, physical therapists work to improve patients' health and assist them in getting back to their normal daily activities. In other trials, it was discovered that PT helped patients become more physically active throughout the entire CR process [31]. Generally speaking, evidence from various studies has shown that preoperative and postoperative physiotherapy aids in early functional recovery, decrease hospital stay and mortality rate, and promote shorter hospital stays [32, 33].

## 5. Conclusion

Our findings and their analysis show that PT plays a significant role in encouraging the use of CR and preventing NCDs. The profession will require additional training. To produce more and better-informed PT specialists in this subject, education programmes in that area should be developed and made available by universities. Particularly, Indian PTs need greater knowledge and training on CR. To get more patients into rehabilitation, it is strongly advised that professional healthcare workers, patients, and medical students receive additional training on the advantages of CR. By having health insurance providers and the Indian health authorities pay for CR programmes, barriers to entering a CR can be lessened. The other important factor is the experience of the respondents which might vary from a graduate-level practitioner to a doctorate-level practitioner.

### 5.1. Limitations

The major limitation of the study was to obtain appropriate responses from the respondents who received our questionnaire. The other important factor is the experience of the respondents which might vary from a graduate-level practitioner to a doctorate-level practitioner.

### 5.2. Future Recommendation

Further research studies can be performed to overcome the barriers identified in this study and find methods to ensure and promote patient adherence.

## Figures and Tables

**Table 1:** Demographic characteristics.

Demographic characteristics	*n*	%
Highest qualification		
BPT	61	48.0
MPT	62	48.8
Ph.D.	4	3.1
Type of organization		
Educational/research institute	61	48.0
Private clinic	0	0
Hospital	61	48.0
Home services	0	0
NGO/community service	5	3.9
Region		
Rural	10	7.9
Suburban	31	24.4
Urban	86	67.7
Gender		
Female	98	77.2
Male	29	22.8
Zone of practice		
Central India	3	2.4
North India	1	0.8
East India	1	0.8
Northeast India	0	0
West India	81	63.8
South India	41	32.3
Participation in cardiac rehabilitation		
Yes	5	3.9
No	122	96.1

**Table 2:** Knowledge of physiotherapists in India about cardiopulmonary rehabilitation.

Knowledge	Physiotherapists *N* = 127 *n* (%)
Knowledge about CR level	
Very poor	7 (5.5%)
Poor	7 (5.5%)
Medium	58 (45.6%)
Good	45 (35.4%)
Excellent	10 (7.8%)
Knowledge about CR content	
Very poor	11 (8.6%)
Poor	7 (5.5%)
Medium	53 (41.7%)
Good	46 (36.2%)
Excellent	10 (7.8%)

**Table 3:** Attitude of physiotherapists towards cardiopulmonary rehabilitation.

Attitude	Physiotherapists *N* = 127 *n* (%)
Do you agree that cardiopulmonary rehabilitation in India is effective?	
Agree	48 (37.8%)
Disagree	79 (62.2%)
Do you agree that access for an outpatient canter is an added value in India?	
Agree	122 (96.2%)
Disagree	5 (3.9%)
Do you agree that cardiopulmonary rehabilitation could increase the quality of life and lifestyle of the patient?	
Agree	121 (95.4%)
Disagree	6 (4.7%)
Do you agree that cardiopulmonary rehabilitation could change patient behaviours postsurgery or suffering from cardiovascular diseases?	
Agree	119 (94.2%)
Disagree	1 (6.2%)

**Table 4:** Practice of physiotherapists in India regarding cardiopulmonary rehabilitation.

Practice	Physiotherapists *N* = 127 *n* (%)
What kind of patients do you consider suitable for cardiopulmonary rehabilitation after discharging from the hospital?	
Cardiac patients	
Yes	237 (73.6%)
No	79 (24.5%)
COPD patients	
Yes	271 (84.1%)
No	45 (14%)
Postsurgery patients	
Yes	126 (39.1%)
No	190 (59%)
Muscular dystrophy patients	
Yes	83 (25.7%)
No	233 (72.3%)
Cancer patients	
Yes	72 (22.96%)
No	244 (77.83%)
Diabetic patients	
Yes	70 (21.73%)
vNo	246 (76.4%)
Patients suffering from pulmonary diseases	
Yes	248 (77.1%)
No	68 (21.1%)
Transplantation patients	
Yes	157 (48.7%)
No	159 (49.4%)
Obese patients	
Yes	57 (17.7%)
No	259 (80.4%)
Do you think it would be difficult for a physiotherapist to refer patients to CR in in India?	
Yes	102 (80.3%)
No	25 (19.6%)
Who should we take the initiative to initiate cardiopulmonary rehabilitation in India?	
Insurance companies	
Yes	51 (40.5%)
No	76 (59.8%)
Physiotherapists	
Yes	91 (71.8%)
No	36 (28.3%)
Doctors	
Yes	85 (66.8%)
No	42 (33%)
Policy providers	
Yes	61 (48%)
No	66 (51.9%)
How many patients did you treat suffering from cardiopulmonary diseases during the last month at your work place?	
0	108 (84.9%)
1-2	9 (7%)
3–10	8 (6.2%)
11–20	2 (1.5%)
>21	0 (0%)
How many patients did you treat suffering from cardiopulmonary diseases during the last month at patients' home?	
0	112 (88.4%)
1-2	8 (6.2%)
3–10	6 (4.7%)
11–20	1 (0.7%)
>21	0 (0%)

**Table 5:** Barriers faced by physiotherapists to refer patients to cardiopulmonary rehabilitation in India.

Barriers	Physiotherapists *N* = 127 *n* (%)
Do you observe any barriers when patients are referred from physicians/primary care providers to start a rehabilitation program?	
Yes	82 (64.9%)
No	45 (35.4%)
What kind of barriers do you face?	
More skills are needed in India	
Yes	71 (55.9%)
No	56 (44%)
More specialists are needed in India	
Yes	54 (42.8%)
No	73 (57.48%)
More equipped centres are needed in India	
Yes	40 (31.6%)
No	87 (68.5%)
Lack of interest in cardiopulmonary rehabilitation	
Yes	18 (13.8%)
No	119 (93.7%)
Cardiopulmonary rehabilitation would benefit the patient	
Yes	19 (15.2%)
No	108 (85%)
Cardiopulmonary rehabilitation would not change the patient behaviour	
Yes	19 (15.2%)
No	108 (85%)
Price of the cardiopulmonary rehabilitation program will be a barrier	
Yes	54 (42.8%)
No	73 (57.4%)
No covering by insurances companies and NSSF will be a barrier	
Yes	41 (31.9%)
No	86 (67.7%)
Not enough endorsement by physicians will be a barrier	
Yes	31 (24.2%)
No	96 (75.6%)
Others kinds of barriers:	
Yes	42 (33.2%)
No	85 (66.9%)

**Table 6:** Role played by physiotherapists in the promotion of cardiopulmonary rehabilitation in India.

Role of physiotherapist	Physiotherapists *N* = 127 *n* (%)
Role of physiotherapist is:	
To discuss the benefits of a healthy and active lifestyle with the patients	
Agree	127 (100%)
Disagree	0 (0%)
To promote the prevention of cardiovascular diseases	
Agree	127 (100%)
Disagree	0 (0%)
To encourage physical activity in their clinical practice on a daily basis	
Agree	126 (99.2%)
Disagree	1 (0.7%)
To be confident when giving advice to the patient on physical activity	
Agree	120 (94.5%)
Disagree	7 (5.5%)
To be active, healthy to act as a model for their patients	
Agree	109 (85.6%)
Disagree	18 (14.1%)
To assess patients, i.e., BMI, make screening of the cardiovascular risk factors	
Agree	119 (94%)
Disagree	8 (6.2%)
To prescribe exercise counselling according to the patient assessment beyond exercise therapy	
Agree	68 (53.8%)
Disagree	59 (46.4%)
To assess exercises capacity, i.e. via 6-minute walk test	
Agree	75 (58.8%)
Disagree	52 (41%)
To give diet/nutritional counselling	
Agree	74 (58.2%)
Disagree	53 (41.73%)
To give smoking cessation counselling	
Agree	71 (56.1%)
Disagree	56 (44%)
To make patient education to help them cope with their illness and improve their health-related quality of life	
Agree	121 (95.2%)
Disagree	6 (4.7%)
To give psychological management during treatment	
Agree	61 (47.9%)
Disagree	66 (451.9%)
To make recommendations posttreatment on exercises	
Agree	169 (96%)
Disagree	1 (0.6%)

**Table 7:** Support from physiotherapists in India for different kinds of forms of cardiopulmonary rehabilitation.

Support	Physiotherapists *N* = 127 *n* (%)
Support to cardiopulmonary rehabilitation:	
Support for inpatient cardiopulmonary rehabilitation	
Yes	118 (92.8%)
No	9 (7%)
Support for outpatient cardiopulmonary rehabilitation	
Yes	119 (93.4%)
No	8 (6.2%)
Support for home-based cardiopulmonary telerehabilitation	
Yes	92 (72.2%)
No	35 (27.5%)

## Data Availability

The data used to support the findings of this study are available from the corresponding author upon request.

## Supplementary Materials

Supplementary Materials The questionnaire of the study was included in the supplementary materials.
