# Supplementary material for: Knowledge, Attitude, and Practice of Physiotherapists about Cardiac Rehabilitation Program Adherence among Patients Discharged from the Hospital after Cardiac Surgery in India
Source: ScientificWorldJournal. 2024 May 17;2024:8825476. doi: 10.1155/2024/8825476 (PMC11126335; doi:10.1155/2024/8825476)
Supplement: Supplementary Materials — The questionnaire of the study was included in the supplementary materials. [file 8825476.f1.docx]

**A supplement to study about questionnaires**

**Title- QUESTIONNAIRE ON “KNOWLEDGE, ATTITUDE AND PRACTICE OF PHYSIOTHERAPISTS ABOUT CARDIAC REHABILITATION PROGRAM ADHERENCE AMONG PATIENTS DISCHARGED FROM HOSPITAL AFTER CARDIAC SURGERY”**

About the questionnaire-

A criterion of 60% and above was kept to include the question in the questionnaire of the study. All experts graded 4 or 5 for the 27 questions, which ranked greater than 60%. The validity of the questionnaire was done using CVI (content validity index). Item-content validity index (I-CVI) and scale-content validity index (S-CVI) were calculated for the item pool. The S-CVI was calculated by taking the average I-CVI and dividing it by the total number of items. The S-CVI was 0.97. From the values obtained, we could conclude that the questionnaire had achieved a satisfactory level of content validity. Knowledge based questions were 13-19 and attitude based questions were 8-12. Now research says that Phase II CR can be given after discharge at home or back at hospital under supervision. At 19 th question we divided where questions 20-25 who worked at home based and 20-21 was framed for hospital based CR. These questions 20-25 were a mix of knowledge , attitude and barriers.

**Screening Questionnaire**

We need to make sure that you are eligible to take part in this project.

Please read the following statements carefully. You should not take part in this project if you

- Do not provide physiotherapy to cardiac patients.
- Do not practice in India

Do any of these statements describe you?

- None of these statements describes me (I am eligible to take part)
- Yes, at least one of these statements describes me (I am not eligible to take part)

**Demographics**

1. What is your highest level of education?
   - BPT
   - MPT
   - PhD
2. What best describes the type of organization you work for?

- Educational/Research Institute
- Private Clinic
- Hospital
- Home Services
- NGO/Community Service

1. In which region do you work?

- Rural
- Sub-urban
- Urban

1. What is your gender?

- Female
- Male
- Others
- Prefer not to say

1. In which zone of India do you practice?

- Central India
- North India
- East India
- North-East India
- Western India
- South India

1. Do you practice Cardiac Rehabilitation?

- Yes
- No

1. How long have you been practicing Cardiac Rehabilitation?

- Less than 3 months
- 3-6 months
- 6-12 months
- 1-2 years
- 2-5 years
- More than 5 years

1. In your opinion what is the importance of cardiac rehabilitation?

- Not important
- Somewhat important
- Important
- Very important

Is it a common practice to recommend cardiac rehabilitation in the place where you practice?

Yes

- No

1. Have you specifically received any formal training for cardiac rehabilitation other than your curriculum?

- Yes
- No

1. Do you have a multidisciplinary team at your hospital for Cardiac patient’s care?

- Yes
- No

1. Do you actively participate as a team member?

- Yes
- No

1. How many phases of Cardiac Rehabilitation are there?

- 1
- 2
- 3-4

1. Where is the phase I Cardiac Rehabilitation given?

- At the hospital
- At home
- It is not required

1. Do you think that the patients should be followed up after discharge?

- Yes
- No

1. In your opinion is Phase II Cardiac Rehabilitation equally important as Phase I?

- Yes
- No

1. Does Phase III Cardiac Rehabilitation require follow up?

- Yes
- No

1. Do you think Cardiac Rehabilitation is a life-long program?

- Yes
- No

1. How do you practice Phase II Cardiac Rehabilitation?

- As home program
- Supervised at hospital after Phase I
- Community based
- We don’t follow up patients after discharge

If as home program:

1. How do you assess patient adherence to the exercise protocol?

- Call the patient to the hospital once/twice a week
- Visit the patient at his/her home
- Call the patient on phone
- Ask the patient to maintain a diary
- Do not assess patient adherence

1. What measures do you use to increase the intensity of exercise/ decide the target intensity?

- Use Rate of perceived exertion
- Use Heart rate
- Use Metabolic equivalent values (METs)
- Combination of the above methods

1. How do you ensure the safety of the patient?

- Leg pain as patient self-reported to stop
- Breathlessness as patient self-reported to stop
- Give target heart rate
- Any other

1. Do you teach patients to use a perceived exertion scale?

 Yes

 No

1. How many minutes of physical activity is ideally advised to the patients?

 10-20 mins/day

 20-30 mins/day

 30-40 mins/day

1. What is the minimum days a patient should exercise per week?

 2 days

 3 days

 5 days

If Phase II is supervised at the hospital:

20. How is the patient turnover?

- Poor (<30% of the patients follow up)
- Fair (30% to 70% of the patients follow up)
- Good (> 70% of the patients follow up)

21. If the patient turnover is low what do you think are the reasons for the same?

- Non referral
- Lack of awareness
- Lack of support from the family
- Transport issue
- Monetary issue
- Any other reason
